# Rescue of Cyclic AMP Mediated Long Term Potentiation Impairment in the Hippocampus of Mecp2 Knockout (*Mecp2^-/y^*) Mice by Rolipram

**DOI:** 10.3389/fncel.2016.00015

**Published:** 2016-02-03

**Authors:** Saju Balakrishnan, Marcus Niebert, Diethelm W. Richter

**Affiliations:** Institute for Neuro and Sensory Physiology, University of GöttingenGöttingen, Germany

**Keywords:** memory, plasticity, patch clamp, forskolin, adenylyl cyclase

## Abstract

Rett syndrome (RTT) patients experience learning difficulties and memory loss. Analogous deficits of hippocampal plasticity are reported in mouse models of RTT. To elucidate the underlying pathophysiology, we studied long term potentiation (LTP) at the CA3 to CA1 synapses in the hippocampus in acute brain slices from WT and *Mecp2^-/y^* mice, by either activating cAMP dependent pathway or using high frequency stimulation, by means of patch clamp. We have observed that, the NMDA channel current characteristics remain unchanged in the *Mecp2^-/y^* mice. The adenylyl cyclase (AC) agonist forskolin evoked a long lasting potentiation of evoked EPSCs in WT CA1 neurons, but only minimally enhanced the EPSCs in the *Mecp2^-/y^* mice. This weaker potentiation in *Mecp2*^-/^*^y^* mice was ameliorated by application of phosphodiesterase 4 inhibitor rolipram. The hyperpolarization activated cyclic nucleotide gated channel current (*I*_h_) was potentiated to similar extent by forskolin in both phenotypes. Multiple tetanus induced cAMP-dependent plasticity was also impaired in the *Mecp2*^-/^*^y^* mice, and was also partially rescued by rolipram. Western blot analysis of CA region of *Mecp2*^-/^*^y^* mice hippocampus revealed more than twofold up-regulation of protein kinase A (PKA) regulatory subunits, while the expression of the catalytic subunit remained unchanged. We hypothesize that the overexpressed PKA regulatory subunits buffer cAMP and restrict the PKA mediated phosphorylation of target proteins necessary for LTP. Blocking the degradation of cAMP, thereby saturating the regulatory subunits alleviated this defect.

## Introduction

Patients with Rett syndrome (RTT) have severe mental retardation. Amir et al. (1999) discovered that the majority of RTT cases result from mutations in the X-linked MECP2 (methyl-CpG-binding protein 2) gene richly expressed in the neurons. The Mecp2 protein binds to methylated cytosine on the genomic DNA through a methyl-CpG-binding domain (MBD) crucial to the gene’s function (Shahbazian et al., 2002; Zhao et al., 2015). The primary function of Mecp2 is thought to be the repression of gene transcription, although, it can also act as a transcriptional activator (Chahrour et al., 2008). Intact Mecp2 function is pivotal to the normal development of nervous system and its absence or mutation can lead to defective dendritic structure, synapse formation and decreased size of the neurons (Gao et al., 2015; Rietveld et al., 2015).

A mouse model for RTT, which shows typical symptoms of the disease, was developed by deletion of the MECP2 gene. Male mice (*Mecp2*^-/^*^y^*) are preferred over female mice for experiments, due to the ubiquitous lack of Mecp2 and similar etiology in individual male mice. The disease phenotype in male mice manifests at approximately 4 weeks of age. Reduced cognitive ability of RTT patients is mirrored in the *Mecp2*^-/^*^y^* mice by the lack of neuronal network modulation such as LTP that underlie learning and memory (Moretti et al., 2006). Asaka et al. (2006) have proposed that a cause for this is the impairment of NMDA receptor function due to a switch of the NR2A to NR2B subunits. In addition, Weng et al. (2011) have described a saturation of LTP in a RTT mouse model, which could be reversed by the NMDA antagonist Memantine.

cAMP synthesis is essential for inducing and maintaining LTP in hippocampal CA1 neurons (Huang et al., 1994; Otmakhova et al., 2000; Otmakhov et al., 2004). The drugs raising cAMP levels can modulate glutamatergic neurotransmission (Greengard et al., 1991) and evoke LTP (Frey et al., 1993; Nguyen et al., 1994; Huang and Kandel, 1995; Bolshakov et al., 1997). In addition, it is shown that inhibition of AC or PKA by antagonists, decrease tetanic stimulation induced LTP in the CA1 neurons (Frey et al., 1993; Otmakhova et al., 2000). Besides, the mice carrying mutated AC I and VIII exhibit deterioration of long term memory (Wong et al., 1999).

It is unknown whether the lack of LTP in *Mecp2*^-/^*^y^* mouse is due to a malfunction of the cAMP pathway, and if, AC-PKA mediated neuronal plasticity is affected in RTT. An indication was deduced from brainstem neurons of the *Mecp2*^-^*^/y^* mouse, that they show a reduced cAMP elevation in response to forskolin and cytosolic calcium elevation (Mironov et al., 2011). Therefore, we focused on the role of different enzymes in the cAMP signal pathway with regard to synaptic plasticity in the *Mecp2*^-^*^/y^* mice and age matched wild type. Our results show that LTP mediated by adenylyl cyclase pathway is defective in the *Mecp2^-/y^* mouse due to the uncoupling of downstream signaling by PKA.

## Materials and Methods

### Preparation of Brain Slices

Heterozygous female *Mecp2* gene knockout mice, [B6.129P2(C)-*Mecp*^2tm-1-1Bird^ (Guy et al., 2001)] were obtained from Jackson Laboratories (Bar Harbor, ME, USA) and crossbred with wild type C57BL/6J males. Symptomatic hemizygous males (*Mecp2*^-^*^/y^*) and the male wild type littermate (*Mecp2*^+^*^/y^*) were used for the experiments during the 6th week of life. All animals were used in accordance with the recommendations of the European Commission (No. L358, ISSN 0378-6978), and experimental procedures were approved by the Committee for Animal Research, Gottingen University. The mice were housed (4–5 littermates per cage) on a 12 h light/dark cycle. For slice preparation, the animals were anesthetized with isoflurane and the brains were quickly immersed in ice cold buffer containing (mM) 110 choline chloride, 25 NaHCO_3_, 25 D-glucose, 11.6 Sodium ascorbate, 7 MgSO_4_, 3.1 sodium pyruvate, 2.5 KCl, 1.25 NaH_2_PO_4_, and 0.5 CaCl_2_ (Shepherd and Svoboda, 2005). Coronal slices (350 μm thickness) of whole brain containing the hippocampus were prepared using Leica VT 1200s Vibroslicer and were stored in artificial cerebrospinal fluid (ACSF) containing (mM): NaCl (126), KCl (3), NaH_2_PO_4_ (1.2), NaHCO_3_ (25), glucose (15), MgSO_4_ (2), and CaCl_2_ (2) and continuously bubbled with carbogen (95% O_2_, 5% CO_2_). The slices were allowed to recover from the slicing procedure for 1 h at room temperature. Afterward they were transferred to a submerged chamber under an upright microscope and superfused with ACSF (flow rate of 2–3 ml/min) at room temperature.

### Electrophysiology

#### Patch Clamp

Hippocampal CA1 pyramidal neurons were visually identified under an upright microscope (BX51, Olympus Optical) with a 40X water-immersion objective that was equipped with infrared differential interference contrast (IR DIC) illumination. Neurons were patch clamped with pipettes of 3–4 MΩ resistance, when filled with a solution containing (mM): K-gluconate (110), KCl (5), HEPES (50), EGTA (0.005), MgSO_4_ (4), ATP (4), GTP (0.2), phosphocreatine (9), and pH to 7.4 with 1 M KOH. Whole cell current recordings were made with the EPC-10 amplifier (HEKA Elektronik, Germany). The series resistance ranged from 10 to 20 MΩ and was not compensated. Recordings showing more than 10% fluctuation in series resistance were discarded from the analysis. Currents were acquired at 10–25 kHz and low pass filtered at 2–5 kHz. EPSCs were evoked by stimulating the Schaffer collateral inputs to CA1 neurons with currents of 10–120 μA, using extracellular Teflon coated platinum electrode and square pulse stimulator equipped with isolated constant current unit (Grass technologies, USA). EPSC amplitudes were measured at a holding potential (*V*_h_) of -70 mV unless specified.

#### Measurement of Long Term Potentiation (LTP)

Fifty micromolar forskolin was applied to evoke EPSC potentiation. Alternatively, LTP was evoked by three 1 s long pulse trains at 100 Hz (0.1 ms pulse width), combined with a switch of the *V*_h_ to -10mV, separated by a 10 min inter-stimulus interval. A 10 min baseline was obtained before the application of LTP inducing protocols. The extend of the potentiation was measured at the end of the experiment, in comparison to the baseline amplitude. Stimulation intensity was adjusted to evoke similar baseline EPSC amplitudes in WT and *Mecp2*^-^*^/y^*.

#### Measurement of *I*_h_ and NMDA Currents

*I*_h_ currents were measured in response to a hyperpolarizing voltage step from -70 to -140 mV in ‘isolation buffer’ as described in Bickmeyer et al. (2002). *I*_h_ amplitude was measured at the end of this voltage step. A Cesium based intracellular solution with the following composition (in mM) was used for the measurement of NMDA currents: CsMeSO_3_ (120), NaCl (2.8), HEPES (10), Tetraethyl ammonium chloride (5), EGTA (0.4), Phosphocreatine (5), NaGTP (0.4), MgATP (4). 20 μM CNQX (6-Cyano-7-nitroquinoxaline-2, 3-dione), and 50 μM Picrotoxin were added to inhibit AMPA/Kainate receptors and GABAA receptors for the recording of NMDA I–V relationship. To determine the proportion of AMPA and NMDA components, EPSCs were evoked at –70 mV (AMPA) and +40 mV (NMDA, in presence of 20 μM CNQX and 50 μM picrotoxin). AMPA/NMDA current ratio was obtained by dividing the peak amplitude at a holding potential of -70 mV by the peak amplitude at +40 mV.

### Western Blot Analysis

To analyze the expression of PKA subunits, CA regions of hippocampus (without dentate gyrus) were collected under visual control from animals that were not used for experimental manipulations. Tissue was homogenized in RIPA buffer [20 mM Tris/HCl, pH 7.4, 15 mM NaCl, 10 mM EDTA, 10 mM Iodacetamide, 1% (v/v) Triton X100, 1% (w/v) Na-Deoxycholate, 0,1% (w/v) SDS] supplemented with a protease inhibitor cocktail (Sigma). Protein concentration was determined by Bradford assay and 30 μg of each sample were mixed with Laemmli buffer [20 mM Tris/HCl, pH 6.8, 2 mM EDTA, 2% (w/v) SDS, 10% (v/v) 2-mercaptoethanol, 10% (v/v) glycerol and 0.3% (w/v) bromophenol blue] and boiled for 5 min at 95°C. Proteins were separated using 10% SDS–PAGE and transferred onto a nitrocellulose membrane. The membrane was blocked with 2% w/v BSA/TBS (pH 7.4) for 30 min at room temperature. The regulatory (#3972, Cell Signaling Technology) and catalytic (sc-30667, Santa Cruz) subunits of PKA were detected using respective primary antibodies (Cell Signaling and Santa Cruz, respectively). The antibodies did not differentiate between different subclasses of the subunit types. After washing, appropriate secondary antibodies (IRDye 680LT and 800CW; LI-COR, Lincoln, Nebraska, USA) were used at a dilution of 1:10,000 for 2 h at RT. The visualization of the antigen–antibody reaction was performed using the Odyssey detection system (LI-COR, Lincoln, NE, USA). Expression level differences were determined by comparing fluorescence intensities between WT and *Mecp2*^-^*^/y^* tissue. Statistical significance of the expression was tested independently for regulatory and catalytic subunits by Student’s *t*-test using GraphPad Prism v5.0. For display, expression levels of the regulatory and catalytic subunit of WT were set to 1.

### Protein Kinase A Assay

Hippocampi from both sides were dissected from WT and *Mecp2*^-/^*^y^* mice on their 6th week of life. Care was taken to completely isolate the Ammon’s horn from Dentate Gyrus. Only the Ammon’s horn was used for the assays. The tissues from nine WT and eight *Mecp2*^-/^*^y^* mice were pooled separately. For protein kinase A (PKA) assay, equal weights (50 mg per experiment) of WT or *Mecp2*^-/^*^y^* tissue were separately homogenized in Teflon coated manual homogenizer. Tissue extracts were assayed for PKA mediated phosphorylation of PepTag A1 fluorescent peptide and quantified according to the protocol outlined by Promega for PepTag^®^ Assay (catalog # V 5340) for cAMP dependent PKA. Phosphorylated peptides have a net -1 charge and move to the anode whereas non-phosphorylated peptides retain the +1 charge and move to the cathode. For quantification, respective negatively charged fluorescent bands of WT and *Mecp2*^-/^*^y^* were cut out from the agarose gel (keeping the volume uniform and approximately 250 μl) and transferred to a separate graduated microcentrifuge tube and heated at 95°C until the slice is melted. The volume was made up to 250 μl with distilled water. 175μl each of the hot agarose was transferred to separate tubes, each containing 75 μl of gel solubilization solution, 100 μl of glacial acetic acid and 150 μl of distilled water. The mixture was vortexed and transferred to a 0.5 ml cuvette. The absorbance of the mixture was measured at 570 nm using a spectrophotometer. The number of moles of phosphorylated peptide is computed using Beer’s law and normalized to that of the non-phosphorylated peptide for WT and *Mecp2*^-/^*^y^*. The statistical significance of the difference in phosphorylation activity was tested by Students *t*-test.

### Drugs

All drugs were purchased from Tocris (Bio-Techne, Germany). CNQX to block AMPA, Picrotoxin to inhibit GABA_A_, forskolin to activate AC, SQ 22536 to block AC and rolipram to inhibit PDE4 were dissolved in DMSO. Final concentration of DMSO during the drug applications did not exceed 0.1%.

### Analysis

Statistical significance of Input-output relation was tested by ANOVA. EPSC traces shown in the figures are average of five sequential recordings. Stimulus artifacts were truncated for clarity. Time kinetics of LTP measurements were calculated from the average of normalized amplitudes of the EPSCs from individual experiments. Average amplitudes of the EPSCs at baseline were compared with those obtained at the end of the experiment to calculate the extent of potentiation. Data are given in mean ± SEM of 3–7 cells as indicated. Statistical significance of the LTP data was tested by single, two or paired *t*-test using Origin 8 (Origin lab, Northampton, USA) or GraphPad Prism (GraphPad Software Inc., La Jolla, CA, USA). *P*-value less than 0.05 was considered significant. Power of the sample sizes (minimum 80%) were calculated using Power calculator (Statistical solutions LLC).

## Results

### NMDA Receptor Mediated Current is not Perturbed in *Mecp2^-/y^* Mice

**FIGURE 1:**
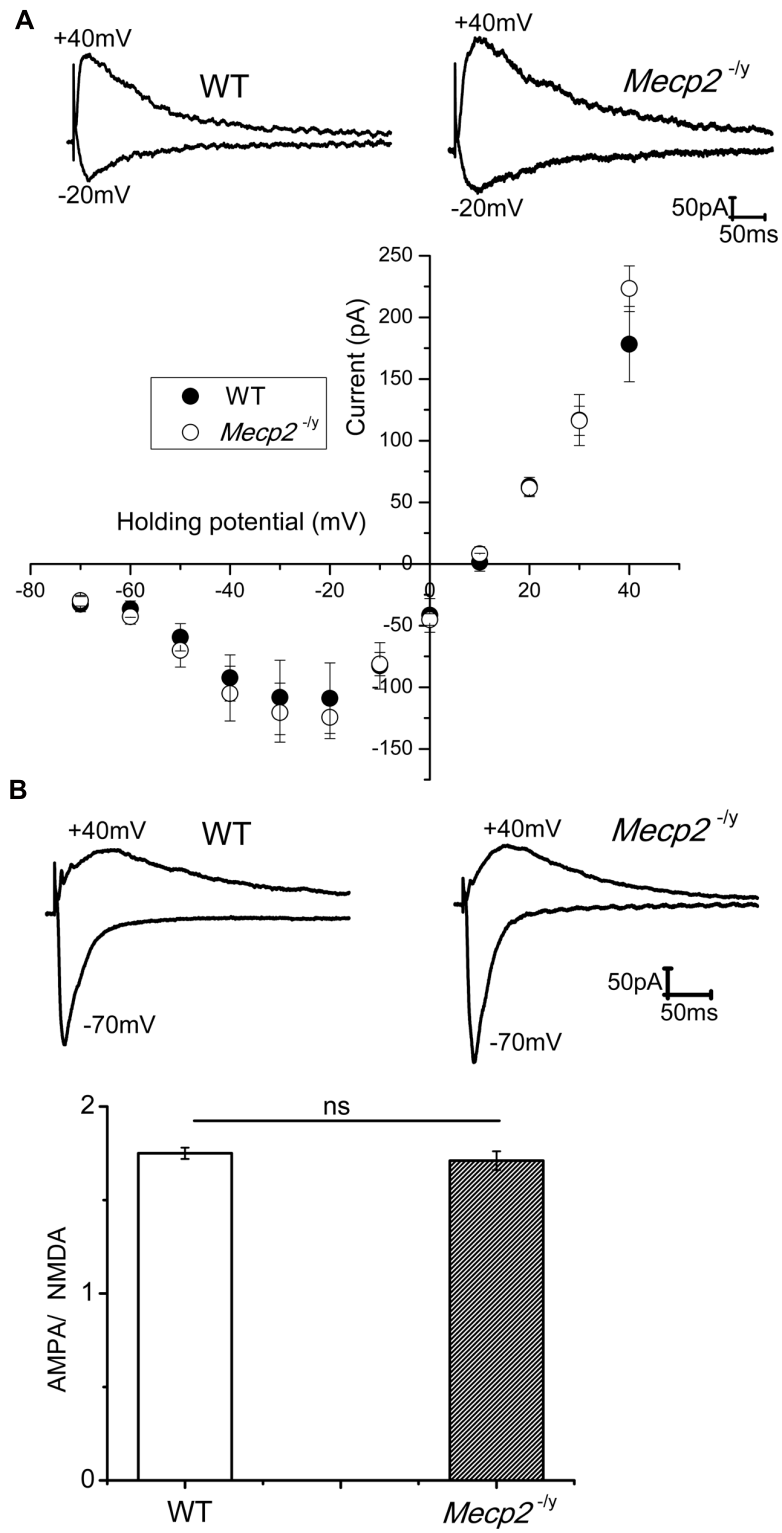
**(A)** NMDA currents had similar amplitudes in WT (*n* = 5) and *Mecp2*^-/^*^y^* (*n* = 7) CA1 neurons. The traces show isolated NMDA currents at -20 mV (inward) and +40 mV (outward) of a WT and *Mecp2*^-/^*^y^* CA1 neurons. The plot represent the IV curve of normalized (to the WT current amplitude at +40 mV) NMDA channel currents from WT and *Mecp2*^-/^*^y^* CA1 neurons evoked at a series of holding potentials from -70 mV to +40 mV in 10 mV steps (filled circles represent WT and open circles denote *Mecp2*^-/^*^y^*). **(B)** Averaged AMPA to NMDA current ratio of WT and *Mecp2*^-/^*^y^* CA1 neurons showing no significant difference in the conductance of these channels between WT and *Mecp2*^-/^*^y^*. The traces indicate the overlaid AMPA (inward) and NMDA (outward) currents of WT and *Mecp2*^-/^*^y^* from a representative experiment.

NMDA channel mediated currents play a crucial role in the induction of neuronal plasticity evoked by both tetanic stimulation and cAMP pathway activation. Even though Asaka et al. (2006) proposed that an altered NMDA subunit expression may underlie the reduced LTP in *Mecp2*^-/^*^y^* mice, physiological evidence is lacking whether the NMDA channel conductance is altered. Therefore, we studied pharmacologically isolated NMDA currents in presence of AMPA and GABA receptor blockers. CA1 neurons were held at varying holding potentials from -70 to +40 mV and NMDA channel mediated synaptic currents were evoked by stimulation of Schaffer collaterals. Stimulation intensity was adjusted to obtain similar amplitude currents at -70 mV in WT and *Mecp2*^-/^*^y^* mice. As shown in **Figure 1A**, the I–V relation of the NMDA currents was not significantly different in WT and *Mecp2*^-/^*^y^* CA1 neurons, neither at negative (at -20 mV: -108.94 ± 28 pA (WT), -124.42 ± 17pA (*Mecp2^-/y^*); *n* = 5, *P* = 0.07, paired sample *t*-test) nor positive (at +40 mV: 178.31 ± 30 pA (WT), 223.3 ± 18pA (*Mecp2*^-/^*^y^*); *n* = 5, *P* = 0.16, paired sample *t*-test) potentials. The AMPA to NMDA ratio of the EPSCs from WT (1.75 ± 0.03) and *Mecp2*^-/^*^y^* (1.71 ± 0.05) was also statistically insignificant (*n* = 5, *P* = 0.06, **Figure 1B**, paired sample *t*-test).

### Forskolin Induced Potentiation of EPSCs is Impaired in the Hippocampus of *Mecp2*^-/^*^y^* Mice

**FIGURE 2:**
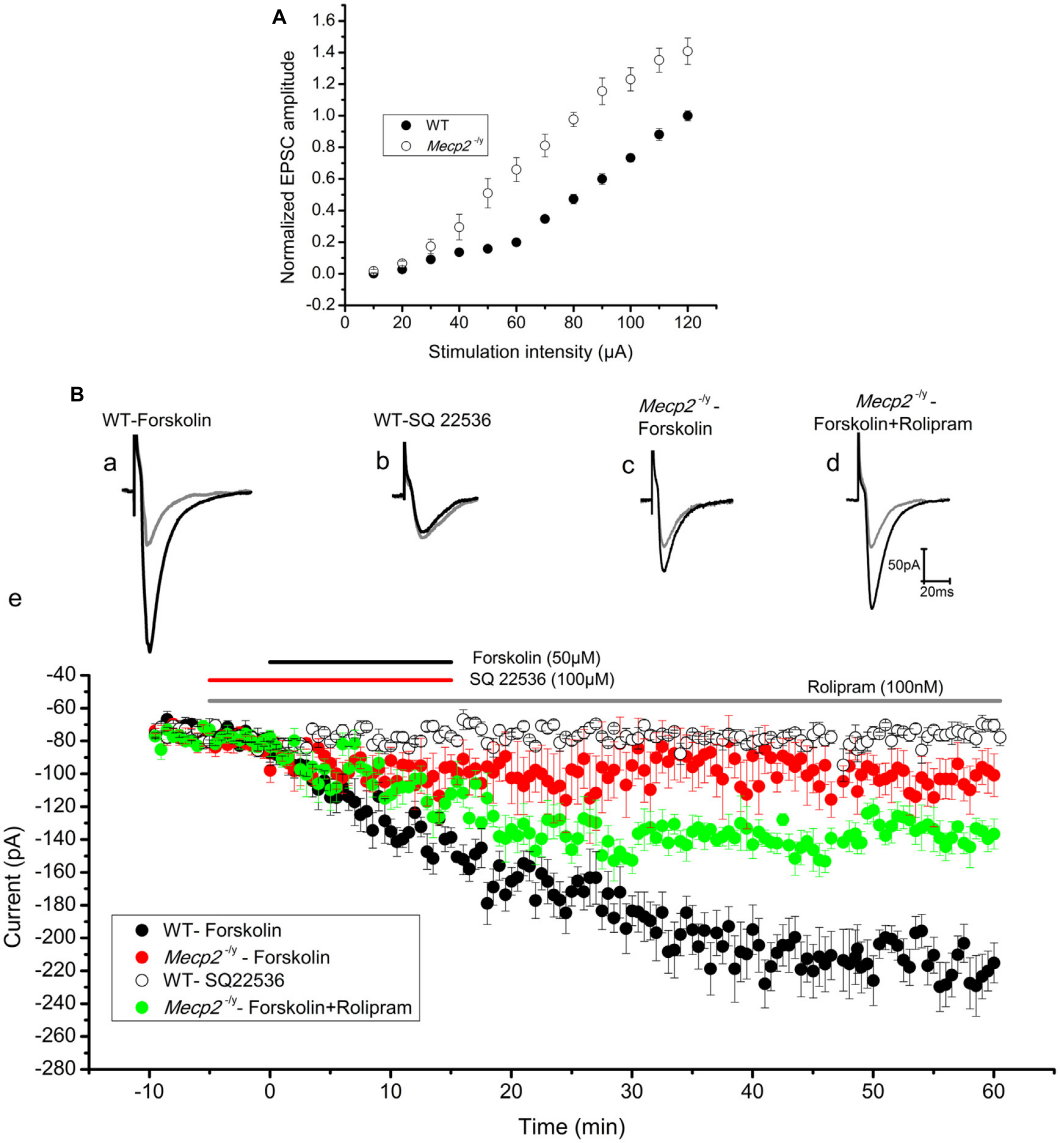
**(A)** Input–output curve of WT (*n* = 5) and *Mecp2*^-/^*^y^* (*n* = 5) CA1 neurons recorded in response to Schaffer collateral stimulation with increasing current steps from 10 to 120 μA. *Mecp2*^-/^*^y^* CA1 neurons were characterized by significantly (*P* < 0.01) increased current ratio to stimulation intensity from 60 to 120 μA. The plot shows EPSC amplitudes normalized to WT value at 120 μA. **(B)** Forskolin enhanced synaptic efficacy in WT but not in *Mecp2*^-/^*^y^* CA1 neurons. Representative EPSC traces are shown in **(Ba)** to **(Bd)** and the time kinetics of EPSC amplitude from each set of experiments are shown in **(Be)**. Gray traces in **(Ba)** to **(Bd)** are the baseline EPSCs and black traces represent EPSC after respective drug applications. **(Ba)** The application of 50 μM Forskolin (black horizontal line in **(Be)**) evoked evident potentiation of the EPSC amplitude in WT (*n* = 8) CA1 neurons. **(Bb)** Addition of adenylyl cyclase inhibitor SQ22536 (100 μM, red horizontal line in **(Be)**) 5 min before Forskolin application blocked EPSC potentiation (*n* = 4). **(Bc)** Forskolin failed to evoke lasting increase in the amplitudes of EPSCs in *Mecp2*^-/^*^y^* mouse (red filled circles in **(Be)**, *n* = 6) CA1 neurons. **(Bd)** Forskolin applied in presence of PDE4 inhibitor rolipram (gray bar in **B**) improved the EPSC potentiation in *Mecp2*^-/^*^y^* significantly (green filled circles in **(Be)**, *n* = 5).

Previous findings show that the extent of electrically induced LTP is lower in the hippocampus of *Mecp2*^-/^*^y^* mouse models (Asaka et al., 2006; Moretti et al., 2006). To evaluate further, we tested forskolin evoked LTP in the hippocampus of *Mecp2*^-/^*^y^* mouse in comparison to WT, through whole cell recordings of EPSCs in CA1 neurons. *Mecp2*^-/^*^y^* neurons displayed comparatively higher input-output relationship to WT neurons [10–40 μA (n.s), 50 μA (*P* < 0.05), 60–120 μA (*P* < 0.01), **Figure 2A**] as previously shown (Janc and Müller, 2014). The LTP experiments are done with a stimulation intensity in the range of 10–40 μA, a range where there is no significant difference in the EPSC amplitudes of WT and *Mecp2*^-/^*^y^* neurons in I/O ratio. After 10 min of control recordings, 50 μM forskolin was bath applied to the slices for 15 min to activate AC, which induced a long-lasting potentiation of EPSC amplitude (2.91 ± 0.07 times the baseline, *n* = 8, *P <* 0.001, single sample *t*-test, **Figure 2Ba** and filled circles in **Figure 2Be**) in the wild type CA1 neurons. This response was completely abolished by inhibiting AC with 100 μM SQ 22536 (1.02 ± 0.07 times baseline, *n* = 4, *P* = 0.95, single sample *t*-test, **Figure 2Bb** and open circles in **Figure 2Be**). Application of SQ 22536 (100 μM) alone did not alter the neurotransmission at basal level stimulation of WT (1.01 ± 0.03 times the baseline, Supplementary Figure S1A, *n* = 5, *P* = 0.13) or *Mecp2*^-/^*^y^* (1.07 ± 0.06 times the baseline, Supplementary Figure S1A, *n* = 5, P = 0.33). CA1 neurons of *Mecp2*^-/^*^y^* mice, however, lacked sustained potentiation of EPSC amplitudes in response to forskolin (1.38 ± 0.17 times the baseline amplitude, *n* = 6, *P* = 0.06, single sample *t*-test, **Figure 2Bc** and filled red circles in **Figure 2Be**). Supplementing forskolin application with the phosphodiesterase 4 inhibitor rolipram (100 nM), which is known to facilitate hippocampal LTP (Barad et al., 1998), enhanced the amplitude of EPSCs significantly in *Mecp2*^-/^*^y^*, although not to WT levels (1.80 ± 0.10 times the baseline amplitude, *n* = 5, single sample *t*-test *P <* 0.01, **Figure 2Bd** and filled green circles in **Figure 2Be**). Perfusion of rolipram (100 nM) alone (without forskolin) did not have significant effect on the amplitude of EPSCs in the WT (0.96 ± 0.1 times the baseline, Supplementary Figure S1B, *n* = 5, *P* = 0.59) and *Mecp2*^-/^*^y^* mice (1.10 ± 0.03 times the baseline, Supplementary Figure S1B, *n* = 5, *P* = 0.22). Moreover, treatment of the slices with forskolin and rolipram did not significantly increase (*P* = 0.97, when compared to LTP induced by forskolin alone, two sample *t*-test) the LTP further in the WT mice (2.99 ± 0.08 times the baseline, *P* < 0.0001, Supplementary Figure S1C, *n* = 5). We compared the expression of forskolin induced LTP in WT and *Mecp2*^-/^*^y^*, by using same stimulus intensity (60 μA) to evoke a similar number of presynaptic inputs. This revealed a difference in the basal amplitude of EPSC in WT and *Mecp2^-^*^/^*^y^*, whereby *Mecp2*^-/^*^y^* CA1 neurons were more excitable than the WT CA1 neurons. Perfusion of 50 μM forskolin invoked a steady increase in the EPSC amplitude and established an LTP of 3.51 ± 0.22 times the baseline (*n* = 5, single sample *t*-test, *P <* 0.001, Supplementary Figure S1D, filled circles) in the WT neurons. Whereas it evoked only a potentiation of 1.29 ± 0.29 times the baseline (*n* = 5, single sample *t*-test, *P <* 0.05) in the *Mecp2*^-/^*^y^* mice.

### cAMP Synthesis and Action in the *Mecp2*^-/^*^y^* Mouse Hippocampus

**FIGURE 3:**
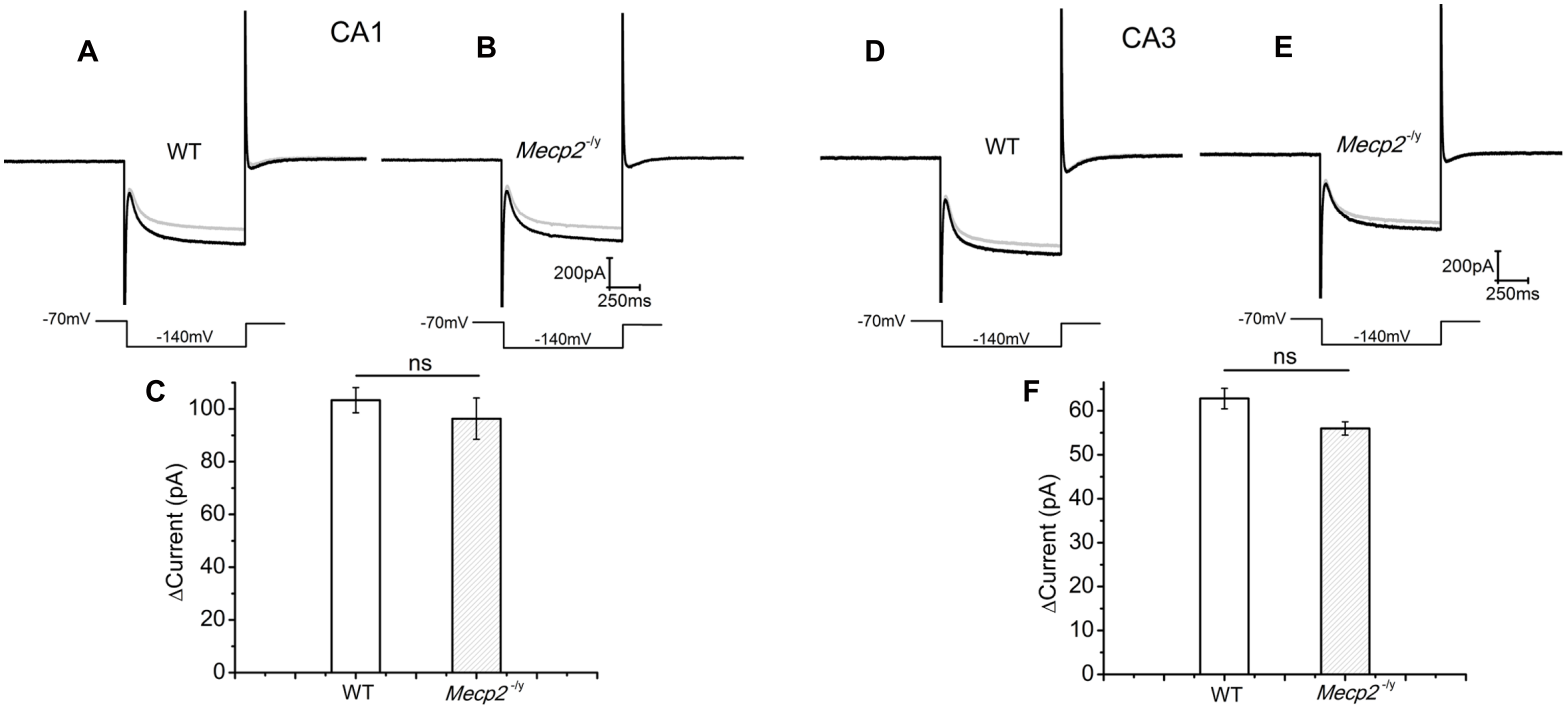
**Forskolin induced enhancement of *I*_h_ was similar in CA1 and CA3 neurons from both genotypes**. *I*_h_ was evoked by a step hyperpolarization from a holding potential of -70 to -140 mV for 1 s as shown below each trace. **(A)** Representative Ih traces from WT CA1 neurons, (*n* = 4) before (gray) and after (black) forskolin application. **(B)** Representative Ih traces from *Mecp2*^-/^*^y^* CA1 neurons (*n* = 4), before (gray) and after (black) forskolin application. **(C)** Averaged current amplitude increase by forskolin in CA1 neurons. **(D)** Representative Ih traces from WT CA3 neurons (*n* = 4), before (gray) and after (black) forskolin application. **(E)** Representative Ih traces from *Mecp2*^-/^*^y^* CA3 neurons (*n* = 4), before (gray) and after (black) forskolin application. **(F)** Averaged current amplitude increase by forskolin in CA3 neurons.

CA1 and CA3 neurons of mice show an *I*_h_ current through the hyperpolarization activated cyclic nucleotide gated (HCN) channels (Vasilyev and Barish, 2002). Drugs that elevate cAMP levels are known to increase the amplitude of *I*_h_ (Gasparini and DiFrancesco, 1999), depolarize the neurons and enhance neurotransmission (Beaumont and Zucker, 2000). Therefore, we used *I*_h_ current amplitude (measured at the end of the voltage step) as readout of the cAMP production (Heine et al., 2002) by forskolin. The measurements showed that 50 μM forskolin caused an *I*_h_ amplitude increase of 103.37 ± 4.76 pA in the WT and 96.33 ± 7.8415 pA in *Mecp2*^-/^*^y^* CA1 neurons (**Figures 3A–C**, *n* = 4, *P* = 0.59, two sample *t*-test). Similarly the increase in the *I*_h_ amplitude (69.07 ± 3.05pA in WT and 60.99 ± 5.2 pA in *Mecp2*^-/^*^y^*) caused by forskolin was not significantly different in WT and *Mecp2*^-/^*^y^* CA3 neurons (**Figures 3D–F**, *n* = 4, *P* = 0.22, two sample *t*-test) of WT and *Mecp2*^-/^*^y^*. Albeit the differences were insignificant, these results show that forskolin is able to activate AC in the CA1 (postsynaptic) and the CA3 (presynaptic) neurons in WT and *Mecp2*^-/^*^y^* to synthesize cAMP and activate a downstream signal process.

### Rescue of the Multiple Tetanus Induced cAMP Dependent LTP in *Mecp2*^-/^*^y^* by PDE4 Blockade

**FIGURE 4:**
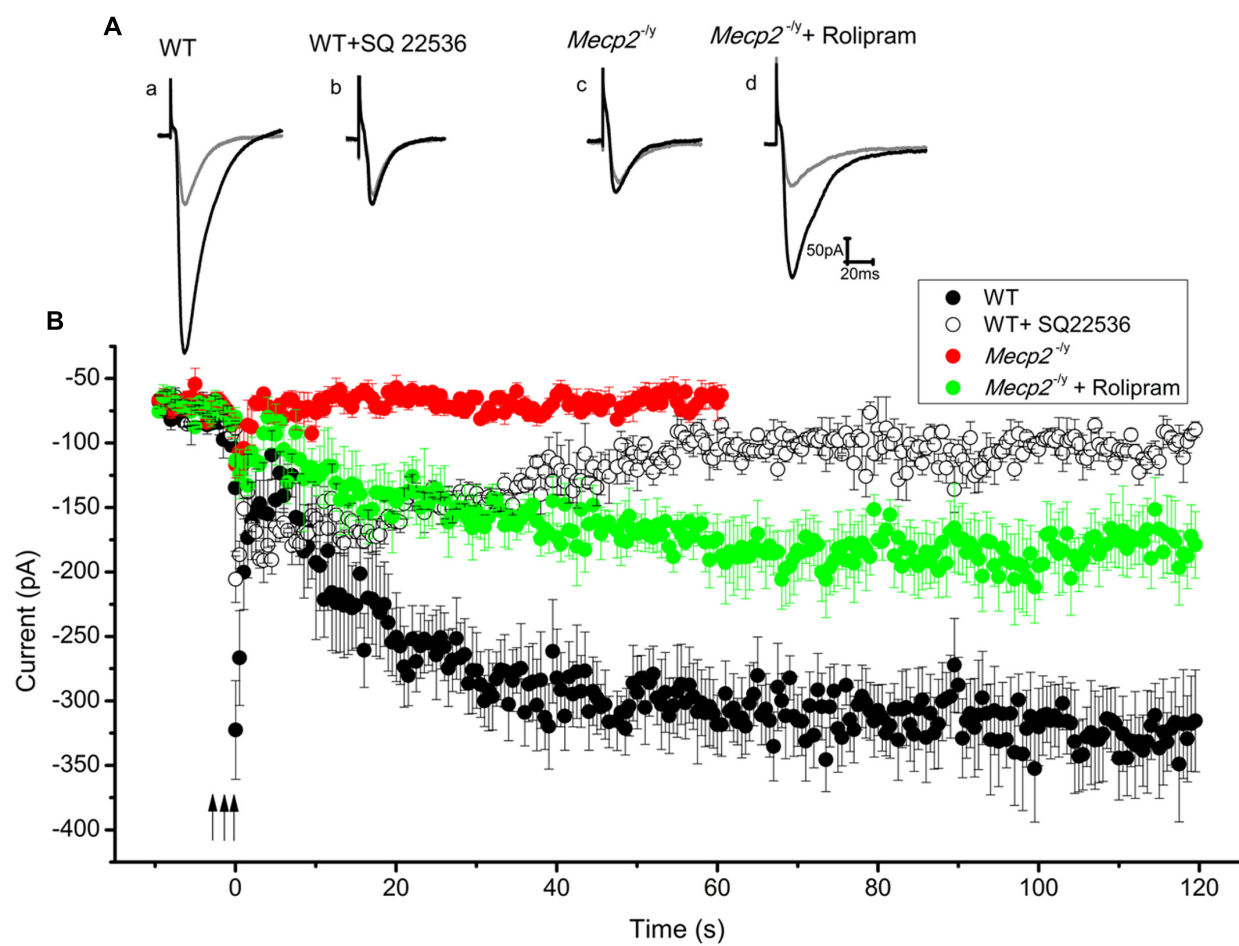
**Three tetanic pulses separated by 10 min were applied to Schaffer collaterals for 1 s each (represented by the arrows) to evoke cAMP dependent LTP**. Traces from individual experiments are shown in **(A)** and the averaged EPSC amplitude time course in B. EPSC amplitude modulation in the WT CA1 neurons is shown in **(Aa)** (gray trace is control and black is during LTP) and filled circles in **(B)** (*n* = 5). Adenylyl cyclase inhibitor SQ 22536 (100 μM) blocked this effect when applied along with the tetanus (for 30 min), **(Ab)** (gray trace is control and black is after SQ22536 treatment) and open circles in **(B)** (*n* = 3). Same tetanic stimulation protocol failed to evoke LTP in *Mecp2*^-/^*^y^* CA1 neurons, **(Ac)** (gray trace is control and black trace represent the averaged EPSCs∼60 min after the tetanus) and red filled triangles in **(B)** (*n* = 5). Treatment of slices with rolipram (for 60 min starting 5 min before the first tetanus) caused a delayed but steady increase in the EPSC amplitude of *Mecp2*^-/^*^y^* CA1 neurons; **(Ad)** (gray is control and black is during LTP) and green filled triangles in **(B)** (*n* = 4).

After forskolin induced LTP induction failed in *Mecp2*^-/^*^y^* mice, we checked whether strong electrical stimulation is more successful. Stimulation with multiple (three) tetani was used to induce the synthesis of cAMP, thereby mediating PKA dependent long lasting potentiation (Frey et al., 1993; Huang and Kandel, 1994). In our experiments, three 100 Hz pulses, each for 1 s, with an inter-stimulus interval of 10 min were applied to the Schaffer collateral inputs of the CA1 neurons. This produced a steady and reproducible LTP for at least 2 h (3.97 ± 0.4 times control amplitude, *n* = 5, *P* < 0.001, single sample *t*-test, **Figure 4Aa** and filled circles in **Figure 4B**) in the WT. The potentiation was blocked by the application of adenylyl cyclase inhibitor SQ22536 (100 μM), applied 5 min before the first until 5 min after the third tetanus (1.24 ± 0.07 times baseline *n* = 3, *P* = 0.86, single sample *t*-test **Figure 4Ab** and open circles in **Figure 4B**). In contrast to the response in WT mice, application of the same tetanic stimulation protocol did not induce significant LTP in the slices from *Mecp2*^-/^*^y^* mice (0.97 ± 0.14 times increase, *n* = 5, *P* = 0.08, single sample *t*-test, **Figure 4Ac** and filled red triangles in **Figure 4B**).

Previous findings have shown that the presence of phosphodiesterase (PDE) inhibitors can strengthen and prolong tetanus induced LTP in CA1 neurons (Barad et al., 1998; Navakkode et al., 2004). Hence it was tested whether the blockage of cAMP degradation during the tetanus could boost the signal transduction and rescue the defective LTP in *Mecp2*^-/^*^y^* mouse CA1 neurons. Perfusion of 100 nM rolipram (from beginning of the acquisition till 60 min after the third tetanus) resulted in a modest increase of the EPSC amplitudes in *Mecp2*^-/^*^y^* CA1 neurons and rescued the LTP to a significant level (2.47 ± 0.25 time baseline, *n* = 4, *P* < 0.05, single sample *t*-test, **Figure 4Ad** and green filled triangles in **Figure 4B**).

### Assay of PKA Activity and Expression Levels

**FIGURE 5:**
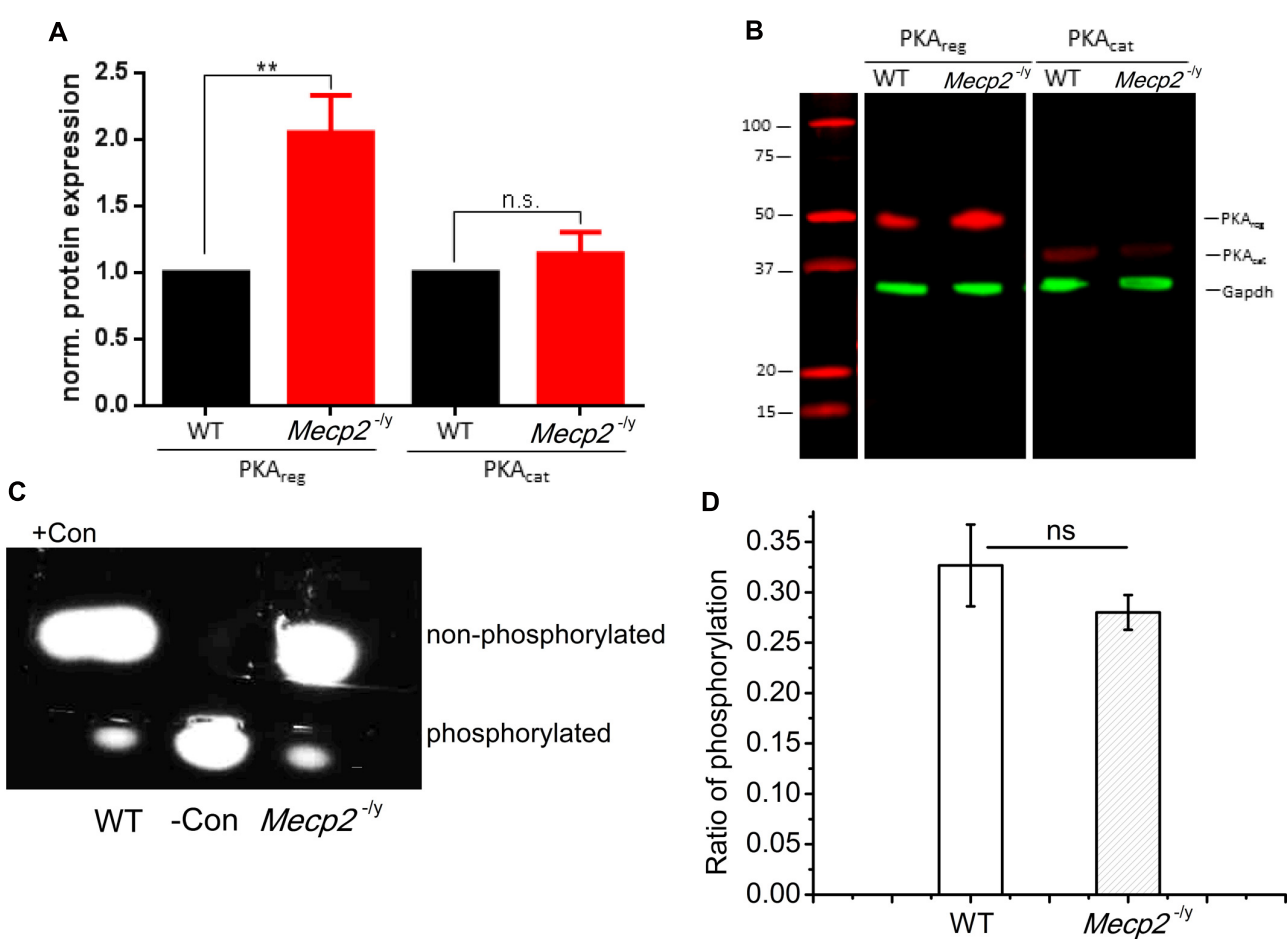
**Quantification of PKA subunit expression in hippocampal tissue of WT and *Mecp2*^-/^*^y^* mice. (A)** Normalized protein expression of the regulatory and catalytic subunit in hippocampal tissue of WT and *Mecp2*^-/^*^y^* mice show a significant (^∗∗^*P* < 0.005) >2-fold upregulation of the regulatory subunits in *Mecp2*^-/^*^y^* tissue. **(B)** Exemplary Western Blot as recorded with the Odyssey system (LI-COR). Samples were run on the same gel and blotted on the same membrane, but PKA subunits (red) were detected separately with GAPDH (green) used as loading control (*n* = 7). **(C)** Analysis of protein kinase A (PKA) activity was tested as the ability to phosphorylate fluorescent test peptides. electrophoresis of the samples from WT and *Mecp2^-/y^*. WT lane indicate the sample where hippocampal extract from WT animals are added and *Mecp2*^-/^*^y^* lane indicate the sample with *Mecp2*^-/^*^y^* hippocampal extract. Con- is the phosphorylated control and Con+ is the non-phosphorylated control. **(D)** PKA phosphorylation activity of WT (*n* = 3) againt *Mecp2*^-/^*^y^* (*n* = 3), is calculated from the ratio of absorbance of phosphorylated bands to that of non-phosphorylated bands at 570 nm.

The electrophysiology results indicate that the adenylyl cyclase function is unimpaired and enhancement of PDE4 activity does improve forskolin and multiple tetanus induced plasticity in *Mecp2^-/y^* mice. Hence the reduction in LTP may result from malfunction of a downstream signaling molecule, viz. PKA. As Mecp2 plays a regulatory role in gene expression, we first checked for the expression of PKA in the CA region of hippocampus. The expression of PKA in the CA layers of hippocampus was analyzed by Western Blot using antibodies against both the catalytic and regulatory subunits of the enzyme. The quantification of bands revealed that, the expression of the regulatory subunits of PKA in the *Mecp2^-/y^* mice (PKA_reg_) was 2.05 ± 0.28 times higher than in WT animals (**Figures 5A,B**, PKA_reg_, *n* = 7, Student’s *t*-test, *P* < 0.005). The catalytic subunit (PKA_cat_) expression levels remained unchanged (**Figures 5A,B**, PKA_cat_, 1.14 ± 0.16, *n* = 7, Student’s *t*-test, *P* = 0.49,) in the *Mecp2*^-/^*^y^* and WT animals.

Tissue extracts of hippocampus from both phenotypes were also analyzed for the PKA enzyme activity, with PepTag assay^®^. The samples were checked for their efficiency in phosphorylating a test peptide provided in the kit. The phosphorylation ability was quantified by spectrophotometry. This analysis revealed that the phosphorylation mediated by PKA in the hippocampal extracts of WT (0.32 ± 0.04, **Figures 5C,D**) and *Mecp2*^-/^*^y^* (0.28 ± 0.02, **Figures 5C,D**) of the test peptide was not significantly different (Two-sample *t*-test*, P* = 0.34). The values are calculated as the ratios of the number of moles of phosphorylated peptide to the non-phosphorylated peptide in each lane for both genotypes.

## Discussion

The cAMP-dependent pathway mediates molecular signaling for memory consolidation (Kandel, 2012). Although it is known that there is LTP defect in RTT (Asaka et al., 2006; Moretti et al., 2006), the underlying causes have not been thoroughly evaluated. The results of this study indicate that NMDA channel activity (current/voltage relationship and AMPA/NMDA ratio) remain unchanged, but the cAMP signaling cascade is defective in the *Mecp2*^-/^*^y^* mouse. Our experiments with forskolin show that direct stimulation of the AC in *Mecp2*^-/^*^y^* mice is unable to induce the long lasting enhancement of neuronal currents of CA1 neurons. Similarly multiple tetani induced LTP was also not expressed in the *Mecp2*^-/^*^y^* mice. Both forms of plasticity could be partially rescued by PDE blocker rolipram.

Direct activation of AC with forskolin is known to enhance glutamatergic currents in the CA1 neurons and PDE inhibition is proven to potentiate this effect further (Chavez-Noriega and Stevens, 1992). The potentiation of EPSC amplitudes elicited in WT slices shown in **Figure 2** is indeed due to AC activity as the specific inhibitor SQ 22536 is able to block it. The absence of forskolin mediated potentiation of EPSCs in the *Mecp2*^-/^*^y^* mice could be due to the reduced activation of AC resulting in lower cAMP synthesis. This was checked by studying the HCN channel currents (*I*_h_) directly influenced by cAMP. Analysis of the efficiency of AC stimulation to modulate *I*_h_ current clearly shows that forskolin is able to activate AC in WT and *Mecp2*^-/^*^y^* mouse hippocampus equally. This observation proves that cAMP production in these neurons was unaltered and cAMP was able to activate a downstream signaling event. *I*_h_ in CA3 neurons (presynaptic) from WT and *Mecp2*^-/^*^y^* expressed similar enhancement in response to forskolin, which confirms that the lack of potentiation in *Mecp2*^-/^*^y^* is not due to a non-responsive *I*_h_ current. These observations lead us to conclude that the cAMP synthesis is intact in *Mecp2*^-/^*^y^* mouse and downstream signaling pathways to AC are analyzed.

Protein kinase A is the major effector in the cAMP signaling cascade, which strengthens the synaptic transmission by phosphorylation of synaptic proteins (Esteban et al., 2003; Bonanomi et al., 2005; Menegon et al., 2006). Postsynaptic PKA activation is specifically shown to be critical for the development and maintenance of different phases of LTP (Blitzer et al., 1995; Nguyen and Kandel, 1997; Otmakhova et al., 2000; Duffy and Nguyen, 2003). We suspected that a change in PKA enzyme function may cause the reduced LTP in *Mecp2*^-/^*^y^* animals. We selected CA1/CA3 regions of hippocampus for PKA activity assay because both presynaptic and postsynaptic PKA activity is important in modulating synaptic communication. We assayed for PKA enzyme activity in WT and *Mecp2*^-/^*^y^* samples, but did not find any significant change, which indicates that the defect in the forskolin mediated potentiation is not due to non-functional PKA. Our Western blot data showed only minor changes in the expression of PKA catalytic subunits in the *Mecp2*^-/^*^y^* mice, justifying the result of the PKA activity assay. However, it revealed a greatly increased expression of the regulatory subunits, which pointed to an excess of free regulatory PKA subunits in the cytoplasm. Such abundant free regulatory subunits will bind cAMP and buffer cAMP levels to restrict its availability for PKA activation. This conclusion is consistent with the reports that the expression of dominant-negative mutants of cAMP binding sites in the regulatory PKA subunit, that are insensitive to cAMP, renders the enzyme functionless (Mellon et al., 1989; Hammerschmidt et al., 1996; Willis et al., 2011; Yang et al., 2014). Hence, cAMP-PKA pathway in *Mecp2*^-/^*^y^*, to be functional, would require a much larger cAMP level to evoke the same effect as in WT animals. Such a reduction of PKA activity would explain the observed defect in the LTP of *Mecp2*^-/^*^y^* CA1 cells. Additionally, PKA localization near AC is shown to be critical in determining the PKA activity and its mediation of LTP at the CA1 region (Kim et al., 2011). cAMP microdomains are usually restricted around AC by the action of PDEs, and colocalization of AC and PKA overcomes this restriction and maximizes the chances of cAMP binding to PKA. We speculate that an overexpression of the regulatory subunit might disrupt this arrangement.

Barad et al. (1998) and Navakkode et al. (2004) state that inhibition PDE4 and enrichment of cAMP life has a positive effect on the induction and maintenance of tetanus induced LTP. Similarly, it is shown by Kim et al. (2011) that inhibiting PDE activity can indeed rescue LTP defect caused by the disruption of PKA anchoring. Our experiments with rolipram application prove that the defective LTP in *Mecp2*^-/^*^y^* animals could be rescued by cAMP enhancement. This also indicates that the LTP restrain in *Mecp2*^-/^*^y^* could result from a lack of cAMP availability due to buffering by upregulated PKA regulatory subunits. Inhibition of cAMP hydrolysis, thereby increasing the availability of cAMP, saturated the regulatory subunits and drove the PKA catalysis efficiently. This is also corroborated by the results on PKA enzyme activity, where a saturating amount of cAMP could drive the phosphorylation of target proteins in the tissue extracts from both phenotypes.

## Conclusion

The above findings suggest that there are malfunctions of the cAMP signaling cascade in the *Mecp2*^-/^*^y^* mice. The expression of the regulatory subunit of PKA is significantly upregulated, leading to increased buffering of cAMP and limiting the catalytic function of the enzyme. This defect can be attenuated with the pharmacological enhancement of cAMP availability in the cells by blocking its degradation with rolipram. Further experiments are required to uncover additional defective molecular candidates in the cAMP-PKA signal cascade, eliciting pathophysiology. *In vivo* experiments are required to confirm that cAMP enrichment can indeed ameliorate the memory related symptoms in *Mecp2*^-/^*^y^* mice.

## Author Contributions

SB did the electrophysiology, PKA activity assay, analysis and wrote the manuscript. MN did the PKA Western blot experiment and analysis. DWR acquired the funding for the project and contributed to the design and analysis of the experiments.

## Conflict of Interest Statement

The authors declare that the research was conducted in the absence of any commercial or financial relationships that could be construed as a potential conflict of interest.

## Supplementary material

FIGURE S1 **(A)** Adenylyl cyclase inhibitor 100 μM SQ 22536 (bar) did not affect EPSC amplitude (normalized to baseline, *n* = 5 each) in the WT and *Mecp2^-^*^/^*^y^* CA1 neurons. Inset shows the representative EPSCs during the baseline (gray) and at the end of the measurement (black) from WT (left) and *Mecp2^-^*^/^*^y^* (right). **(B)** PDE4 inhibitor rolipram (100 nM) did not evoke EPSC potentiation by itself in the WT and *Mecp2*^-/^*^y^* CA1 neurons. Inset traces, WT (left) and *Mecp2*^-/^*^y^* (right), represent the EPSCs before (gray) and at the end of the rolipram treatment (black). The plot shows the EPSC amplitudes normalized to baseline (*n* = 5). **(C)** Supplementing with rolipram (100 nM) did not significantly enhance the forskolin induced LTP further in the WT CA1 neurons (black filled circles, *n* = 5, 2.99 ± 0.08 times the baseline; *P* = 0.97, when compared to LTP induced by forskolin alone) displayed by the time kinetics of normalized EPSC amplitude in the graph. The potentiation induced by forskolin in the WT is shown for comparison (open circles, 2.91 ± 0.07 times the baseline, *n* = 8). Traces represent the EPSCs before (gray) and at the end of the experiment. **(D)** LTP induced by forskolin in WT and *Mecp2*^-/^*^y^* CA1 with a stimulus intensity of 60 μA to recruit similar number of presynaptic inputs. Forskolin application produced evident potentiation in the WT (filled circles) but was much reduced in the *Mecp2*^-/^*^y^* (open circles). Inset shows representative EPSCs from each phenotype.
